# Feline Origin of Rotavirus Strain, Tunisia, 2008

**DOI:** 10.3201/eid1904.121383

**Published:** 2013-04

**Authors:** Mouna Ben Hadj Fredj, Elisabeth Heylen, Mark Zeller, Imene Fodha, Meriam Benhamida-Rebai, Marc Van Ranst, Jelle Matthijnssens, Abdelhalim Trabelsi

**Affiliations:** Sahloul University Hospital, Sousse, Tunisia (M. Ben Hadj Fredj, I. Fodha, M. Benhamida-Rebai, A. Trabelsi);; University of Monastir, Monastir, Tunisia (M. Ben Hadj Fredj, I. Fodha, M. Benhamida-Rebai, A. Trabelsi);; University of Leuven, Leuven, Belgium (E. Heylen, M. Zeller, M. Van Ranst, J. Matthijnssens)

**Keywords:** group A rotavirus, full genome characterization, G6P[9], interspecies transmission, viruses, Tunisia, rotavirus

## Abstract

In Tunisia in 2008, an unusual G6P[9] rotavirus, RVA/human-wt/TUN/17237/2008/G6P[9], rarely found in humans, was detected in a child. To determine the origin of this strain, we conducted phylogenetic analyses and found a unique genotype constellation resembling rotaviruses belonging to the feline BA222-like genotype constellation. The strain probably resulted from direct cat-to-human transmission.

Group A rotaviruses (RVAs) are a leading cause of severe acute gastroenteritis in infants and young children. An infectious RVA virion is a triple-layered icosahedral particle that contains 11 segments of double-stranded RNA (*1*). The outer protein layer is formed by virus capsid protein (VP) 4 (P antigen) and VP7 (G antigen), each of which is used for binomial nomenclature (*1*). At least 27 G genotypes and 35 P genotypes have been identified (*2*). Globally, only 6 G/P-genotype combinations are of epidemiologic relevance to humans: G1P[8], G3P[8], G4P[8], G9P[8], and G12P[8], which are typically found in combination with a Wa-like genotype constellation (I1-R1-C1-M1-A1-N1-T1-E1-H1), and G2P[4], which is found in combination with a DS-1-like genotype constellation (I2-R2-C2-M2-A2-N2-T2-E2-H2) (*3*).

Certain G genotypes rarely encountered in humans are commonly associated with RVA strains from animals (*4*). For example, G6 RVA strains are occasionally detected in humans but are a common genotype in cattle (*4*). Complete genomes have been determined for 11 human G6 RVA strains: 7 G6P[14], 2 G6P[9], 1 human–animal reassortant G6P[6], and 1 unique G6P[11] (*5*–*9*).

The P[9] genotype is commonly associated with the G3 or G6 genotype and is believed to be typical for feline and canine RVA strains (*4*). A few G3P[9] and G3P[3] RVA strains have been detected in humans, and they are believed to be the result of direct interspecies transmission from cats or dogs to humans, possibly in combination with reassortment (*10*–*13*).

Previously, 2 genotype constellations among feline and canine RVA strains, cat97-like and AU-1-like, were described (*13*). The genotype constellations were G3-P[3]-I3-R3-C2-M3-A9-N2-T3-E3-H6 and G3-P[9]-I3-R3-C3-M3-A3-N3-T3-E3-H3, respectively. Recently, the complete genomes of a feline strain (RVA/cat-wt/ITA/BA222/2005/G3P[9]) and 2 feline-like human RVA strains (RVA/human-wt/ITA/PAI58/1996/G3P[9] and RVA/human-wt/ITA/PAH136/1996/G3P[9]) were shown to possess a distinct genotype constellation, G3-P[9]-I2-R2-C2-M2-A3-N1/N2-T3/T6-E2-H3 (*11*), representing a tentative third feline genotype constellation (BA222-like). This tentative third feline BA222-like genotype constellation is an intriguing genotype mosaic, sometimes possessing Wa-like nonstructural protein (NSP) 2 or NSP3 gene segments and partially resembling the genotype constellation found in RVA strains from cattle and other artiodactyla (*5*,*7*,*10*,*11*). Full-genome sequences of unusual human RVA strains are being analyzed to detect interspecies transmission, reassortment, and evolutionary relationships between human and animal RVAs (*10*,*11*). 

In 2008, during continuous surveillance for human RVA in Tunisia, we identified an unusual G6P[9] strain in an 8-month-old hospitalized child (*14*). To understand the evolution and origin of this unusual strain, RVA/human-wt/TUN/17237/2008/G6P[9] (hereafter referred to as strain 17237), we conducted phylogenetic analyses.

## The Study

The full-length genome sequence of the virus was determined as described (*5*). Primers used for all 11 segments are shown in Technical Appendix Table 1. Multiple sequence alignments and phylogenetic analyses were conducted by using MEGA version 5.05 (www.megasoftware.net). Sequences were deposited in GenBank (accession nos. JX271001–JX271011).

Strain 17237 possessed the unique genotype constellation G6-P[9]-I2-R2-C2-M2-A3-N1-T6-E2-H3. This constellation was compared with that of the human G6P[9] strain Se584, feline/canine-like human RVA strains (KF17, PAH136, PAI58, and 0537), and several animal strains (Table). Strain 17237 shared the same combination of genotypes with human RVA strain PAH136 (*10*) except for VP7 (strain 17237 contained G6 instead G3). Overall, strain 17237 shared 8–10 genotypes with RVA strains possessing the BA222-like genotype constellation and 8–9 genotypes with several bovine or bovine-like RVA strains.

**Table:** Comparison of genomic constellation of group A rotavirus strain RVA/human-wt/TUN/17237/2008/G6P[9] from Tunisia with reference strains*

Strain	Genotype constellation	VP7	VP4	VP6	VP1	VP2	VP3	NSP1	NSP2	NSP3	NSP4	NSP5
RVA/human-wt/TUN/17237/2008/G6P[9]	BA222-like	**G6**	**P[9]**	**I2**	**R2**	**C2**	**M2**	**A3**	**N1**	**T6**	**E2**	**H3**
RVA/human-wt/ITA/PAH136/1996/G3P[9]	BA222-like	G3	**P[9]**	**I2**	**R2**	**C2**	**M2**	**A3**	**N1**	**T6**	**E2**	**H3**
RVA/cat-wt/ITA/BA222/2005/G3P[9]	BA222-like	G3	**P[9]**	**I2**	**R2**	**C2**	**M2**	**A3**	**N1**	T3	**E2**	**H3**
RVA/human-wt/ITA/PAI58/1996/G3P[9]	BA222-like	G3	**P[9]**	**I2**	**R2**	**C2**	**M2**	**A3**	N2	**T6**	**E2**	**H3**
RVA/human-tc/USA/Se584/1998/G6P[9]	BA222-like	**G6**	**P[9]**	**I2**	**R2**	**C2**	**M2**	**A3**	N2	T1	**E2**	**H3**
RVA/human-wt/JAP/KF17/2009/G6P[9]	BA222-like	**G6**	**P[9]**	**I2**	**R2**	**C2**	**M2**	**A3**	N2	T3	E3	**H3**
RVA/human-wt/USA/0537/2002/G3P[9]	BA222-like	G3	**P[9]**	**I2**	**R2**	**C2**	**M2**	**A3**	N2	T1	**E2**	**H3**
RVA/cat-tc/AUS/Cat2/1984/G3P[9]	BA222-like/ cat97-like	G3	**P[9]**	I3	R3	**C2**	M3	**A3**	**N1**	**T6**	E3	**H3**
RVA/human-tc/ITA/PA169/1988/G6P[14]	Bovine-like	**G6**	P[14]	**I2**	**R2**	**C2**	**M2**	**A3**	N2	**T6**	**E2**	**H3**
RVA/human-wt/BEL/B10925/1997/G6P[14]	Bovine-like	**G6**	P[14]	**I2**	**R2**	**C2**	**M2**	**A3**	N2	**T6**	**E2**	**H3**
RVA/human-wt/ITA/111-05-27/2005/G6P[14]	Bovine-like	**G6**	P[14]	**I2**	**R2**	**C2**	**M2**	**A3**	N2	**T6**	**E2**	**H3**
RVA/cow-tc/FRA/RF/1982/G6P[1]	Bovine	**G6**	P[1]	**I2**	**R2**	**C2**	**M2**	**A3**	N2	**T6**	**E2**	**H3**
RVA/cow-tc/VEN/BRV033/1990/G6P6[1]	Bovine	**G6**	P[1]	**I2**	**R2**	**C2**	**M2**	**A3**	N2	**T6**	**E2**	**H3**
RVA/cow-tc/USA/WC3/1981/G6P[5]	Bovine	**G6**	P[5]	**I2**	**R2**	**C2**	**M2**	**A3**	N2	**T6**	**E2**	**H3**
RVA/cow-tc/KOR/KJ19-2/2004/G6P[7]	Bovine	**G6**	P[7]	**I2**	**R2**	**C2**	**M2**	**A3**	N2	**T6**	**E2**	**H3**
RVA/rhesus-tc/USA/PTRV/1990/G8P[1]	Bovine-like	G8	P[1]	**I2**	**R2**	**C2**	**M2**	**A3**	N2	**T6**	**E2**	**H3**
RVA/human-tc/KEN/B12/1987/G8P[1]	Bovine-like	G8	P[1]	**I2**	**R2**	**C2**	**M2**	**A3**	N2	**T6**	**E2**	**H3**
RVA/human-tc/USA/DS-1/1976/G2P[4]	DS-1-like	G2	P[4]	**I2**	**R2**	**C2**	**M2**	A2	N2	T2	**E2**	H2
RVA/human-tc/JPN/AU-1/1982/G3P3[9]	AU-1-like	G3	**P[9]**	I3	R3	C3	M3	**A3**	N3	T3	E3	**H3**
RVA/human-tc/USA/Wa/1974/G1P1A[8]	Wa-like	G1	P[8]	I1	R1	C1	M1	A1	**N1**	T1	E1	H1

*Boldface indicates genotypes that are identical to group A rotavirus RVA/human-wt/TUN/17237/2008/G6P[9]. VP, virus capsid protein; NSP, nonstructural protein.

**Figure 1:**
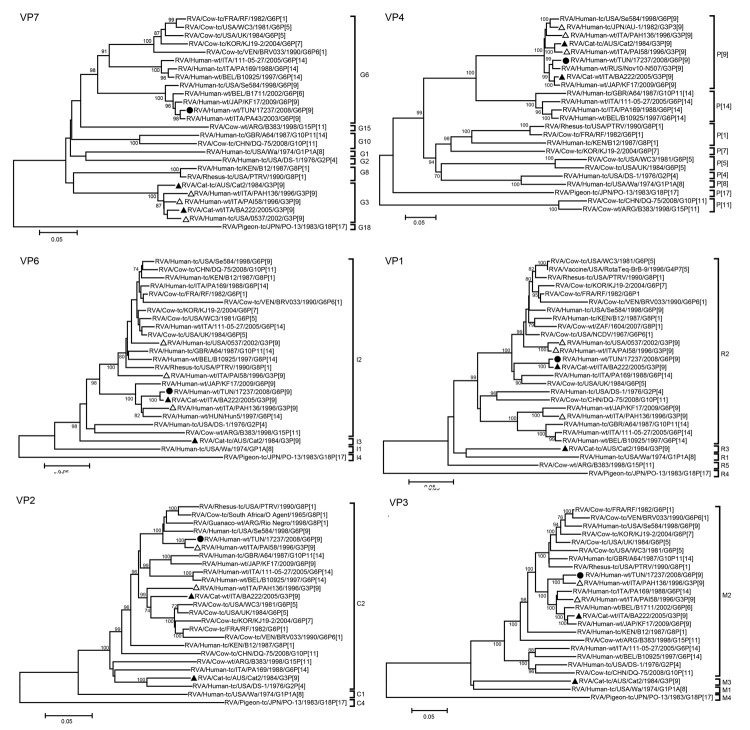
Phylogenetic trees of the full-length nucleotide sequences of the group A rotavirus (RVA) virus capsid protein (VP) 7, VP4, VP6, VP1, VP2, and VP3 genes. Phylogenetic trees were constructed by using the neighbor-joining method with the Kimura 2-parameter method. Bootstrap values (1,000 replicates) >70% are shown. Filled circles indicate strain RVA/human-wt/TUN/17237/2008/G6P[9] from Tunisia; filled triangles indicate feline RVA strains; and open triangles indicate feline/canine-like human RVA strains. GenBank accession numbers of the sequences of reference strains are shown in Technical Appendix Table 2. Scale bars indicate nucleotide substitutions per site.

**Figure 2:**
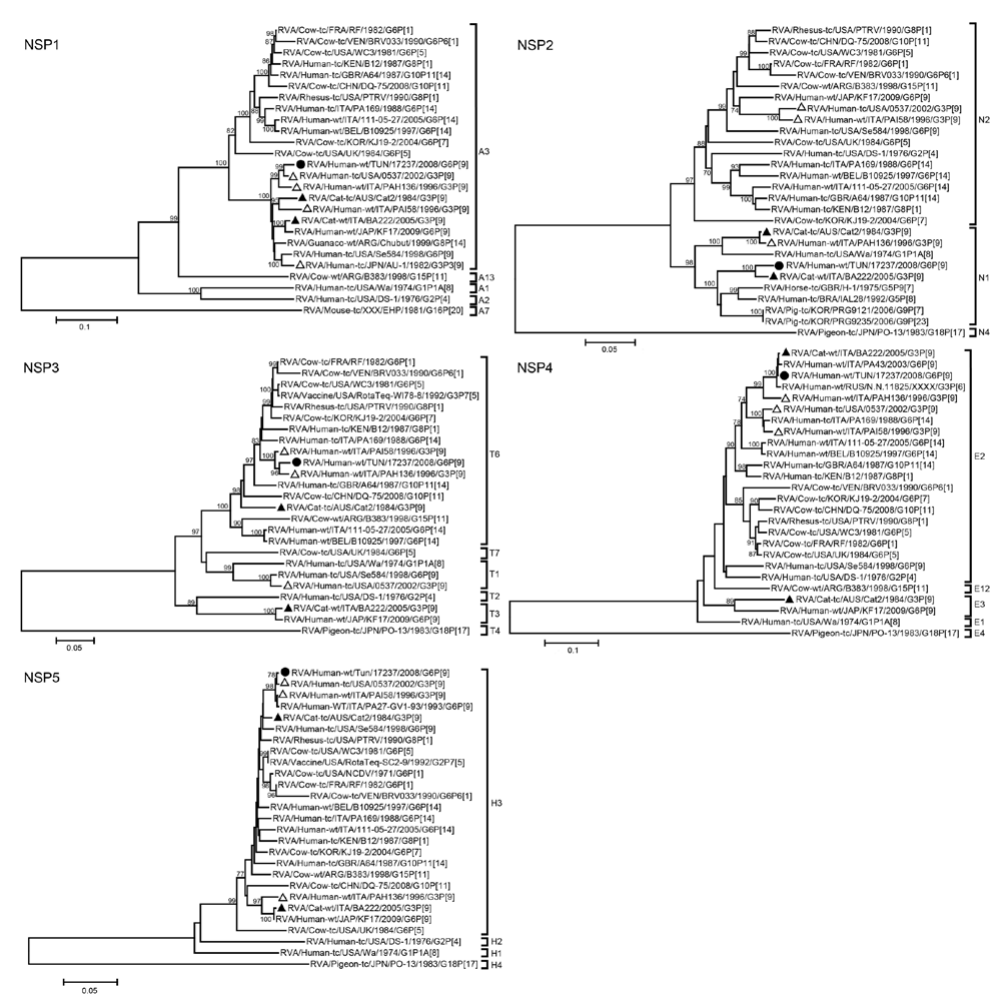
Phylogenetic trees of the full-length nucleotide sequences of the group A rotavirus (RVA) nonstructural protein (NSP) genes. Phylogenetic trees were constructed by using the neighbor-joining method with the Kimura 2-parameter method. Bootstrap values (1,000 replicates) >70% are shown. Filled circle indicates strain RVA/human-wt/TUN/17237/2008/G6P[9] from Tunisia, filled triangles indicate the feline RVA strains, and open triangles indicate the feline/canine-like human RVA strains. GenBank accession numbers of the sequences of reference strains are shown in Technical Appendix Table 2. Scale bars indicate nucleotide substitutions per site.

Phylogenetic analyses showed that all 11 genome segments of strain 17237 were most closely related to strains of either feline-like human or feline origin (Figures 1, 2). Strain 17237 clustered most closely with RVA/human-wt/ITA/PA43/2003/G6P[9], RVA/human-wt/JAP/KF17/2009/G6P[9], and RVA/human-wt/BEL/B1711/2002/G6P[6] strains, all of which are suspected to have at least a partial animal (bovine-like or feline-like) origin (*6*,*7*). The P[9] genome segment was most closely related to RVA strains RVA/human-wt/RUS/Nov10-N507/2010/G3P[9], BA222, and KF17. The VP1, VP6, NSP2, and NSP4 genome segments of strain 17237 were closely related to BA222, clustering in the R2, I2, N1, and E2 genotypes, respectively. This G3P[9] feline RVA strain BA222 is believed to have a common origin with animal RVA strains and RVA strains that are zoonotically transmissible to humans (*11*). The NSP2 gene segment of strain 17237 clustered in the N1 genotype and was distantly related to typical human Wa-like RVA strains. The VP2, NSP3, and NSP5 gene segments were closely related to RVA/human-wt/ITA/PAI58/1996/G3P[9]. The VP3, NSP1, and NSP3 genome segments clustered closely with RVA/human-wt/ITA/PAH136/1996/G3P[9]. NSP1 and NSP5 clustered closely with RVA/human-wt/USA/0537/2002/G3P[9]. These 3 human strains (PAI58–96, PAH136–96, and 0537) are believed to be of feline origin and possess a BA222-like genotype constellation.

## Conclusions

The genome constellation of strain 17237 is similar to that of strains belonging to the tentative feline BA222-like genotype constellation (Table). It has been speculated that several of these BA222-like RVA strains resulted from multiple reassortment events among RVA strains originating from different hosts (cattle, other ruminants, humans, cats, dogs) (*10*,*11*). However, a recent article speculates that this genotype constellation, although reminiscent to bovine-like RVA strains, might represent a true feline genotype constellation (*12*). 

Our results support this hypothesis in 2 ways. The first source of support comes from the fact that BA222-like RVA strains have been detected on several continents: Europe (Italy), North America (United States), Asia (Japan), and now Africa (Tunisia) (*7*,*10*). RVA strains with this BA222-like genotype constellation are much more likely to circulate in a certain host species rather than result from distinct multiple reassortment events in each of the above-mentioned countries. The second source of support comes from the fact that our phylogenetic analyses confirmed that each of the 11 gene segments of strain 17237 was more closely related to BA222-like RVA strains than to bovine or bovine-like RVA strains. This finding strengthens the hypothesis that each of the BA222-like RVA strains did not result from individual multiple reassortment events but rather that this genotype constellation now circulates (most likely in cats) around the world and might have resulted from >1 reassortment events in the more distant past. 

To further support or refute this hypothesis, more complete genomes must be determined from RVA strains from cats and dogs. Moreover, because P[9] is believed to be typical for feline/canine RVA strains, it would be intriguing to determine whether this strain could persist in the human population and could become competitive with already established P genotypes in humans. The recently emerged human G9 RVA strain is believed to have originated from pigs and to have become established in the human population as the fifth major human RVA genotype, after multiple genome reassortment events with typical human Wa-like RVA strains (*15*). 

The unusual G6P[9] RVA strain 17237 most likely resulted from direct interspecies transmission from a cat to a human. Interspecies transmission increases potential for spread of unusual and uncommon RVA strains. The findings of this study highlight the need for continuous monitoring of RVA strains and timely recognition of novel or rare genotypes. Continued surveillance of RVA strains in industrialized and developing countries, and in humans and animals, will provide more insights into interspecies transmission processes of RVAs. In turn, this information could help determine how the introduction of novel genes might affect the evolution of the RVA populations that infect humans.

Technical Appendix List of primers used for amplification and sequencing of the whole-genome of strain RVA/human-wt/TUN/17237/2008/G6P[9] and GenBank accession numbers of the sequences of reference strains used in the phylogenetic analysis.
