# Supplementary material for: Feline Origin of Rotavirus Strain, Tunisia, 2008
Source: Emerg Infect Dis. 2013 Apr;19(4):630–4. doi: 10.3201/eid1904.121383 (PMC3647418; doi:10.3201/eid1904.121383)
Supplement: Technical Appendix — List of primers used for amplification and sequencing of the whole-genome of strain RVA/human-wt/TUN/17237/2008/G6P[9] and GenBank accession numbers of the sequences of reference strains used in the phylogenetic analysis. [file 12-1383-Techapp-s1.pdf]

Article DOI: http://dx.doi.org/10.3201/eid1904.121383

Feline Origin of Rotavirus Strain, Tunisia

Technical Appendix

Table 1. Primers used for amplification and sequencing of the whole genome of group A rotavirus strain RVA/human-wt/TUN/17237/2008/G6P[9] from Tunisia, 2008

Gene	Primer name	Primer sequence
VP1	Gen_VP1Fb	5′-GGC TAT TAA AGC TRT ACA ATG GGG AAG -3′
	Gen_VP1Rb	5′-GGT CAC ATC TAA GCG YTC TAA TCT TG -3′
	MG6_VP1_447F	5′-TGC AGT TAT GTT CTG GTT GG -3′
	Hosokawa_VP1_2587R	5′-ACG CTG ATA TTT GCG CAC -3′
	LAP_VP1_1200F	5′-GCT GTC AAT GTC ATC AGC -3′
	Gen_VP1_2417R	5′-GCT ATY TCA TCA GCT ATT CCY G -3′
	30-96_VP1_3163F	5′-GGA TCA TGG ATA AGC TTG TTC TG -3′
	26097_VP1_269R	5′-GCG TTA TAC TTA TCA TAC GAA TAC G -3′
VP2	Gen_VP2Fc	5′-GGC TAT TAA AGG YTC AAT GGC GTA CAG -3′
	Gen_VP2Rbc	5′-GTC ATA TCT CCA CAR TGG GGT TGG -3′
	26097_VP2_458F	5′-AGT TGC GTA ATA GAT GGT ATT GG -3′
	B1711_VP2_2112R	5′-GCA ATT TTA TCT GAG GCA CG -3′
	NCDV_VP2_1868F	5′-AGG ATT AAT GAT GCA GTG GC -3′
	LAP_VP2_2543F	5′-GAC ATC AAA TCT TAC CTT CAC TG -3′
	260-97_VP2_345R	5′-GAC TCT TTT GGT TCG AAA GTA GG -3′
	FR5_VP2_23F	5′-TAC AGG AAA CGT GGA GCG -3′
	260-97_VP2_744R	5′-GTACTCTTTGTCTCATTTCCGC -3′
	Gen_VP2_2739Ra	5′-TAC AAC TCG TTC ATG ATG CG -3′
VP3	Gen_VP3_24F	5′-TGY GTT TTA CCT CTG ATG GTG-3′
	Gen_VP3_2584R	5′-TGA CYA GTG TGT TAA GTT TYT AGC-3′
	NCDV_VP3_2026R	5′-CAT GCG TAA ATC AAC TCT ATC GG -3′
	MG6_VP3_488F	5′-GCA GCT ACA GAT GATGAT GC -3′
	B10925_VP3_2416F	5′-ACA ATC GAG AAT GTT CAT CCC -3′
	TUN1_VP3_167R	5′-TTT CTA CTG CAG CTA TGC CAG-3′
VP4	LAP_VP4_788F	5′-CCT TGT GGA AAG AAA TGC-3′
	VP4_2348-2368Re	5′-GGT CAC ATC TTA AAA TAG ACA G -3′
	TUN1_VP4_2101F	5′-CAG ACT CAC CAG TTT TAT CAG C -3′
	TUN1_VP4_199R	5′-ACT GGT AAG TTG AGT GAG GTG -3′
VP6	Gen_VP6F	5′-GGC TTT WAA ACG AAG TCT TC -3′
	Gen_VP6R-RC	5′-GGT CAC ATC CTC TCA CT -3′
	E403_VP6_1192F	5′-TGG CTT CCA TTA GAA GCA TGC -3′
	TUN1_VP6_229R	5′-TGC ATT ACG CGC AGT TTC AAC -3′
VP7	Beg9-deg	5′-GGC TTT AAA AGM GAG AAT TTC CG -3′
	End9-deg	5′-GGC TTT AAA AGM GAG AAT TTC CG -3′
	PA169_VP7_895F	5′-AGA ATG ATG CGA GTG AAT TGG -3′
	TUN1_VP7_250R	5′-AAG GGT CGA TGT CAA AAA CGG -3′
NSP1	Gen_NSP1F	5′-GGC TTT TTT TTA TGA AAA GTC TTG-3′
	Gen_NSP1R-RC	5′-GGT CAC ATT TTA TGC TGC C-3′
	Chu_NSP1_1463R	5′-CAT TCT TCC TAT TCA GTG CTC G -3′
	Chu_NSP1_1310F	5′-ATG GAT GGA AGA GTG CCA AGG -3′
	Chu_NSP1_265R	5′-GGG TTC TTC ATC TAA GAA ACA CC -3′
NSP2	Gen_NSP2F	5′-GGC TTT TAA AGC GTC TCA G-3′
	Gen_NSP2R	5′-GGT CAC ATA AGC GCT TTC -3′
	Au-1_NSP2_951F	5′-CCT GTC AAC TGA TAG AAA GAT GG -3′
	POR_NSP2_132R	5′-ACT TTT GCT GTC AAC ATG C -3′
	D2-25_NSP2_471R	5′-TGA CCA ATA GCT ATC AAC ACA G -3′
NSP3	Gen_NSP3F	5′-GGC TTT TAA TGC TTT TCA GTG-3′
	Gen_NSP3R	5′-ACA TAA CGC CCC TAT AGC-3′
	Au-1_NSP3_519R	5′-ACT TCC ATT TTC TCT TCA ACG A -3′
	TUN1_NSP3_786F	5′-GCC TGA TGA AAT TAA GAC GGA C -3′
NSP4	NSP4F	5′-GGC TTT TAA AAG TTC TGT TCC GAG-3′
	NSP4R	5′-GGT CAC ATC AAG ACC ATT CC-3′
	PA169_NSP4_553F	5′-TGA CTG CAG CAA TGT GAG -3′
	TUN1_NSP4_122R	5′-AAA GAC TGT CAG GAC AGA TGC -3′
NSP5	Gen_NSP5F	5′-GGC TTT TAA AGC GCT ACA G-3′
	Gen_NSP5R	5′-GGT CAC AAA ACG GGA GT -3′
	MG6_NSP5_503F	5′-CGA TGA TTC TGA TAG TGA TGA TGG -3′
	LAP_NSP5_314R	5′-CGT GAT TGT GTT GAT GAA TCC -3′

Table 2. GenBank accession numbers of the sequences of reference strains used in the phylogenetic analysis

Strain	VP7	VP4	VP6	VP1	VP2	VP3	NSP1	NSP2	NSP3	NSP4	NSP5
RF	X65940	U65924	K02254	J04346	X14057	AY116592	M22308	Z21640	Z21639	AY116593	AF188126
WC3	AY050272	AY050271	AF411322	EF560615	EF560616	EF560617	EF990699	EF990700	EF990701	AY050273	EF990702
UK	JF693056	JF693051	JF693053	JF693048	JF693049	JF693050	JF693052	JF693054	JF693055	JF693057	JF693058
KJ19-2	FJ206095	HM988969	HM988973	HM988966	HM988961	HM988963	HM988971	FJ206116	FJ206167	FJ206106	FJ206054
BRV033	U62154	*U62155*	AF317126	EF560612	EF560613	EF560614	EF990703	EF990704	EF990705	AF144804	EF990706
111-05-27	EF554142	EF554140	EF554141	EF554137	EF554138	EF554139	EF554143	EF554144	EF554145	EF554146	EF554147
PA169	EF554131	EF554129	EF554130	EF554126	EF554127	EF554128	EF554132	EF554133	EF554134	EF554135	EF554136
B10925	EF554120	EF554118	EF554119	EF554115	EF554116	EF554117	EF554121	EF554122	EF554123	EF554124	EF554125
Se584	EF672609	EF672605	EF583044	EF583041	EF583042	EF583043	EF672606	EF672608	EF672607	EF672610	EF672611
B1711	AF532202	*EF554085*	*EF554086*	*EF554082*	*EF554083*	EF554084	*EF554088*	*EF554089*	*EF554090*	*EF554091*	*EF554092*
KF17	JF421980	JF421978	JF421979	JF421975	JF421976	JF421977	JF421981	JF421982	JF421983	JF421984	JF421985
PA43	JF793944	*JF793939*	*JF793933*	–	–	–	–	–	–	JF793928	*JF793922*
B383	FJ347116	FJ347114	FJ347115	FJ347111	FJ347112	FJ347113	FJ347117	FJ347118	FJ347119	FJ347120	FJ347121
A64	EF672567	EF672563	EF583020	EF583017	EF583018	EF583019	EF672564	EF672566	EF672565	EF672568	EF672569
DQ-75	GU144587	GU181281	GU384194	GU384192	GU384191	GU384193	GU384195	GU384196	GU384197	GU181282	GU384198
Wa	JX406755	JX406750	JX406752	JX406747	JX406748	JX406749	JX406751	JX406754	JX406753	JX406756	JX406757
DS-1	HQ650124	HQ650119	HQ650121	HQ650116	HQ650117	HQ650118	HQ650120	HQ650123	HQ650122	HQ650125	HQ650126
B12	HM627547	HM627545	HM627546	HM627542	HM627543	HM627544	HM627548	HM627549	HM627550	HM627551	HM627552
PTRV	FJ422138	FJ422134	FJ422136	FJ422131	FJ422132	FJ422133	FJ422135	FJ422139	FJ422137	FJ422140	FJ422141
Cat2	EU708961	EU708959	EU708960	EU708956	EU708957	EU708958	EU708962	EU708963	EU708964	EU708965	EU708966
PAH136	GU296430	GU296426	GU296428	GU296420	GU296422	GU296424	GU296410	GU296412	GU296415	GU296416	GU296418
PAI58	GU296431	GU296427	GU296429	GU296421	GU296423	GU296425	GU296411	GU296413	GU296414	GU296417	GU296419
BA222	GU827411	GU827409	GU827410	GU827406	GU827407	GU827408	GU827412	GU827413	GU827414	GU827415	GU827416
0537	JF805014	*JF805012*	JF805013	JF805010	*JF805011*	*JF805015*	JF805005	JF805006	JF805007	JF805008	JF805009
AU-1	*D86271*	D10970	*DQ490538*	*DQ490533*	*DQ490536*	*DQ490537*	D45244	*DQ490534*	*DQ490535*	*D89873*	*AB008856*
Nov10-N507	*JX088008*	JQ289055	*JQ230092*	–	–	–	–	–	–	–	–
Hun5	*EF554109*	*EF554107*	EF554108	*EF554104*	*EF554105*	*EF554106*	*EF554110*	*EF554111*	*EF554112*	*EF554113*	*EF554114*
1604	*JN831225*	*JN831223*	*JN831224*	JN831220	*JN831221*	*JN831222*	*JN831215*	*JN831216*	*JN831217*	*JN831218*	*JN831219*
NCDV	*JF693034*	*JF693029*	*JF693031*	JF693026	*JF693027*	*JF693028*	*JF693030*	*JF693032*	*JF693033*	*JF693035*	JF693036
Chubut	*FJ347105*	*FJ347103*	*FJ347104*	*FJ347100*	*FJ347101*	*FJ347102*	FJ347106	*FJ347107*	*FJ347108*	*FJ347109*	*FJ347110*
O-Agent	*JF693045*	*JF693040*	*JF693042*	*JF693037*	JF693038	*JF693039*	*JF693041*	*JF693044*	*JF693043*	*JF693046*	*JF693047*
Rio Negro	*FJ347127*	*FJ347125*	*FJ347126*	*FJ347122*	FJ347123	*FJ347124*	*FJ347128*	*FJ347129*	*FJ347130*	*FJ347131*	*FJ347132*
H-1	*AF242393*	*FJ870377*	*AF242394*	*JQ309138*	*JQ309139*	*JQ309140*	*JQ309141*	JQ309142	*JQ309143*	*AF144800*	*JQ309144*
IAL28	*EF672588*	*EF672584*	*EF583032*	*EF583029*	*EF583030*	*EF583031*	*EF672585*	EF672587	*EF672586*	*EF672589*	*EF672590*
PRG9121	*JF796739*	*JF796737*	*JF796738*	*JF796734*	*JF796735*	*JF796736*	*JF796729*	JF796730	*JF796731*	*JF796732*	*JF796733*
PRG9235	*JF796706*	*JF796704*	*JF796705*	*JF796701*	*JF796702*	*JF796703*	*JF796696*	JF796697	*JF796698*	*JF796699*	*JF796700*
N.N.11825	–	–	–	–	–	–	–	–	–	DQ270109	–
PA27-GV1-93	*JF793942*	*JF793937*	*JF793931*	–	–	–	–	–	–	*JF793926*	JF793921
RotaTeq BrB-9	*GU565090*	*GU565088*	*GU565089*	GU565085	*GU565086*	*GU565087*	*GU565091*	*GU565092*	*GU565093*	*GU565094*	*GU565095*
RotaTeq WI78-8	*GU565079*	*GU565077*	*GU565078*	*GU565074*	*GU565075*	*GU565076*	*GU565080*	*GU565081*	GU565082	*GU565083*	*GU565084*
RotaTeq SC2-9	*GU565068*	*GU565066*	*GU565067*	*GU565063*	*GU565064*	*GU565065*	*GU565069*	*GU565070*	*GU565071*	*GU565072*	GU565073
PO-13	D82979	AB009632	D16329	AB009629	AB009630	AB009631	*AB009633*	AB009625	AB009626	AB009627	AB009628
EHP	*U08425*	*U08424*	–	–	–	–	U08423	–	–	*U96336*	–

*Numbers in italic were not used in this study. – indicates that no sequence data were available in GenBank.
